# Measuring and Mapping Alcohol Outlet Environment Density, Clusters, and Racial and Ethnic Disparities in Durham, North Carolina, 2017

**DOI:** 10.5888/pcd18.210127

**Published:** 2021-09-23

**Authors:** Mike Dolan Fliss, Mary E. Cox, John W. Wallace, Matthew C. Simon, Kendall B. Knuth, Scott Proescholdbell

**Affiliations:** 1 University of North Carolina Injury Prevention Research Center, Chapel Hill, North Carolina; 2 North Carolina Division of Public Health, Injury and Violence Prevention Branch, Raleigh, North Carolina; 3 North Carolina Institute for Public Health, University of North Carolina, Chapel Hill, North Carolina; 4 Council of State and Territorial Epidemiologists, Applied Epidemiology Fellowship, Atlanta, Georgia

**Figure:**
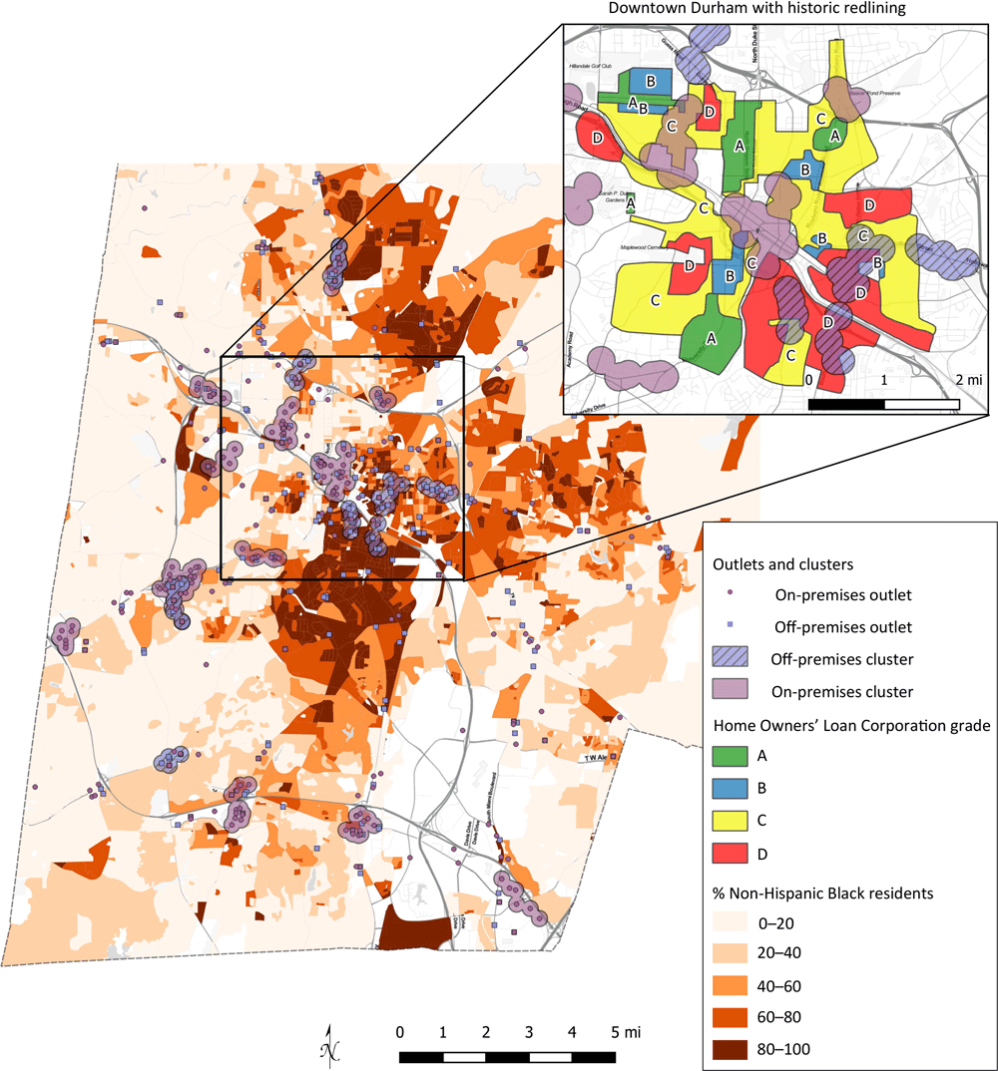
Static display of Durham County, North Carolina’s on- and off-premises alcohol outlets in 2017. Historic redlined areas from the same region are inset, established in 1933 by the Home Owners’ Loan Corporation. Data sources: 2017 American Community Survey 5-year block group estimates, 2013–2017; North Carolina Alcoholic Beverage Control license database.

## Background

Excessive alcohol consumption is responsible for more than 95,000 deaths in the US each year (1). Policies limiting high densities of alcohol outlets (places where alcohol can be sold or consumed) can curb excessive alcohol consumption (2). Denser alcohol environments are associated with multiple chronic disease pathways (3), neighborhood-level social effects (4), and increased rates of alcohol-related morbidities and mortalities, such as from motor vehicle crashes, pedestrian injuries, and various types of violence (5).

Community partners in Durham County, North Carolina, explored the alcohol and tobacco exposure environment during community-wide conversations about gentrification and neighborhood change (6), laying the groundwork for our study. Given the community context and the role of racism as a fundamental cause of health disparities (7), we supplemented the community-led analysis of racial and ethnic disparities with measures of spatial access and distance to the nearest alcohol outlet. After conversations with community partners, we contextualized these metrics by using maps to emphasize spatial associations of historic racial disenfranchisement and present-day alcohol outlet clusters.

Using the Centers for Disease Control and Prevention (CDC) publication, Guide for Measuring Alcohol Outlet Density (8), Zhang et al identified outlet clusters in Atlanta, Georgia, and found that reducing outlet density was associated with reductions in violent incidents (9). We expanded on that study by using alcohol outlet density metrics (spatial access index, minimum distance) to describe racial and ethnic disparities in the alcohol outlet environment in the city of Durham, North Carolina. We report on-premises (eg, sit-down restaurant) and off-premises (eg, liquor store or gas station) results separately because off-premises settings carry unique and increased population health harms (10). Durham is the name of both a North Carolina county and the largest city within that county. Hereafter, Durham refers to the county, except when stated otherwise.

## Data and Methods

We derived alcohol outlet locations in Durham from the North Carolina Alcoholic Beverage Control license database for 2007 through 2017, representing over 165,000 outlet licenses of more than 60 permit types. Permits were filtered to 38 types of alcohol outlets that sell to individuals, excluding catering, shipping, and wholesalers.

Distances between populations and outlets were calculated by Euclidean (ie, straight line) methods, because of the ease of communication and because it is unclear whether relationships of alcohol outlets and populations operate by driving along roads in a smaller, walkable city. Distance-to-nearest-outlet calculations reached outside of county boundaries to maintain accuracy for residents living near borders (eg, when a Durham resident’s nearest outlet was in a neighboring county)*.* A spatial outlet cluster was defined similarly to that in the Zhang study (9) but with parameters modified for a smaller city context. After comparing the Durham context to Atlanta, we defined clusters on the basis of an overlapping 0.15-mile radius (ie, spatial buffer) around outlets active on January 1, 2017. Clusters were those overlapping areas with at least 5 outlets for both premise types. This method yielded 8 off-premises clusters and 12 on-premises clusters in 2017.

Population data were derived from block group estimates of the 5-year 2017 American Community Survey (https://www.census.gov/programs-surveys/acs). These estimates were distributed into smaller US Census block centroids (center point of shape) for increased accuracy in distance calculations using the 2010, 10-year census block and block group population proportions. Combined race and ethnicity data from the US Census Bureau were used to calculate aggregate demographic-specific outlet density measures. We acknowledge limitations to this approach for measuring disparities, including not having distinct categories for some racial and ethnic groups, such as Hispanic populations including Latino speakers of other languages. Average measures of spatial exposure access (inverse distance-to-nearest 7 outlets) and minimum distance were stratified by race and ethnicity. Home Owners’ Loan Corporation (HOLC) redlining maps and spatial polygons for Durham in the 1930s were gathered from the Richmond University Mapping Inequality project (11) to contextualize disparities.

Statistical analysis was performed in R (12) with some spatial visualization completed in QGIS (13); both are free and open-source software.

## Highlights

To measure racial and ethnic disparities in alcohol outlet density, we assessed the transferability of alcohol density research in Durham, North Carolina, by applying techniques previously used in Atlanta, Georgia. Multiple quantitative methods were used to measure distance-to-nearest-outlet, spatial exposure index, and demographics within outlet clusters. Our study contextualizes present-day racial and ethnic disparities with historic disenfranchisement pathways by overlaying 1930s redlining data with alcohol outlet locations and clusters. Results indicate similar disparities in maps of the present-day alcohol outlets and historic racial divides from redlining.

## Action

The demographic characteristics of residents living within alcohol clusters differed from the population of Durham County. Non-Hispanic White residents are 42% of the Durham County population but make up 47% of the on-premises clusters and only 26% of the off-premises clusters. In contrast, Hispanic residents are 14% of the county population and 12% of the on-premises cluster population but 21% of the off-premises cluster population. Non-Hispanic Black residents are 38% of the county population and 33% of the on-premises cluster population but 47% of the off-premises cluster populations (Table).

**Table:** Cluster and County Demographics, Spatial Exposure Index, and Distance-to-Nearest-Alcohol-Outlet in Durham County, North Carolina, 2017^a^

Factor	On-Premises	Off-Premises	Durham County
**Cluster demographics, no. (%)**			
Non-Hispanic White	3,939 (46.9)	2,316 (26.3)	112,697 (42.1)
Non-Hispanic Black	2,724 (32.5)	4,164 (47.3)	36,077 (37.5)
Hispanic	1,008 (12.0)	1,874 (21.3)	100,260 (13.5)
Total^b^	8,391 (100)	8,794 (100)	267,587 (100)
**Distance to nearest outlet, miles**			
Non-Hispanic White^c ^	0.76 (0.74–0.78)	0.74 (0.72–0.76)	—
Non-Hispanic Black^c^	0.66 (0.64–0.69)	0.54 (0.52–0.56)	—
Hispanic^c^	0.59 (0.56–0.63)	0.48 (0.45–0.51)	—
Spatial exposure index, miles			
White non-Hispanic	11.6 (11.2–12.0)	10.1 (9.8–10.4)	—
Non-Hispanic Black	11.1 (10.7–11.5)	12.6 (12.3–12.9)	—
Hispanic	12.9 (12.2–13.6)	13.9 (13.4–14.5)	—

a Demographic data derived from block group estimates of the 2017 5-year American Community Survey (https://www.census.gov/programs-surveys/acs).

b Total includes other races and ethnicities besides the 3 listed, so total counts and percentages do not add to 100.

c Generalized linear model estimates (95% CI) are outside the premises-specific range.

The average distance to the nearest on-premises or off-premises outlet for non-Hispanic Black residents was 0.48 miles, closer than 0.74 miles for non-Hispanic White residents. Hispanic residents’ average distance to outlets was 0.66 miles, closer than 0.76 miles for non-Hispanic White residents. Non-Hispanic Black and Hispanic residents were also exposed to denser off-premises alcohol environments (higher spatial exposure index) than non-Hispanic White residents. Hispanic residents were exposed to denser on-premises environments as well, whereas non-Hispanic White residents experienced a much less dense off-premises environment (Table). Because thresholds of effect or dose–response curves for outlet spatial exposure indexes and health outcomes are not known, it is unclear how much more of a health effect is indicated by an off-premises score of 14 for Hispanic residents, as compared with a score of 13 for non-Hispanic Black residents, or a score of 10 for non-Hispanic White residents. Hispanic residents had the greatest spatial exposure indices for both on-premises and off-premises outlets.

Adverse effects of alcohol uniquely burden communities of color (14). Partial explanations might be multigenerational effects of planning and zoning, resource distribution, neighborhood investment decisions, and access to and use of health care. The effects of historical redlining, a widespread practice in the 1930s of denying home loans and underinvesting in Black neighborhoods, might still contribute to health disparities observed in communities of color. Although evidence is mounting on the relationship of redlining to present-day health disparities, some authors suggest it is understudied (15). During the 1930s and 1940s, banks drew maps of major cities for the purpose of loan planning. Maps were labeled in colors, with some areas grade A (best, traditionally colored in green), grade B (still desirable, colored in blue), grade C (declining, colored in yellow), and grade D (hazardous, in red). We followed this traditional color scheme on our map. 

HOLC typically marked Black neighborhoods as grade D. This systematized a process of denying housing loans in Black neighborhoods, which blocked Black residents’ access to capital and equity building through homeownership. This perpetuated a cycle of lower wealth accumulation in Black communities, underinvestment by businesses and government, and racial segregation. Redlining, therefore, could be expected to both lead to a present-day concentration of Black residents (if the area had not been recently gentrified) and unique vulnerabilities to undesirable land use patterns and businesses.

Visual inspection of Durham’s HOLC redlining maps shows the largest off-premises outlet clusters in D-grade areas of primarily Black residents (Figure). Structural racial disenfranchisement from the 1930s is still spatially associated with present-day alcohol outlet clustering in Durham. Historic, local-level maps may help interested community groups and policy makers understand how the now-outdated planning processes might have contributed to racial and ethnic disparities in alcohol environments today. Cluster-specific calculations may be combined with person-centered measures to tell a broader story about alcohol outlet exposure and racial and ethnic disparities.

Although novel behavior-level interventions exist (16,17), the Community Preventive Services Task Force (18) has recommended evidence-based interventions, including increasing both the price of alcohol and dram shop (eg, bars, pubs, taverns) liability laws, that may be applied at the population level. Population-level surveillance of alcohol outlet density may inform local interest groups, policy makers, and others interested in preventing excessive alcohol use and racial and ethnic disparities related to the availability and accessibility of alcohol. Combining maps and measures of present-day disparity and historical spatial associations may promote deeper, more meaningful discussion among groups. Redlining map archives of many large US cities are freely available online (11).

We encourage researchers not only to calculate overall alcohol outlet density but also to stratify density and disparity calculations by on-premises restaurant districts and off-premises settings. If these settings are combined, calculations may unintentionally hide meaningful disparities in outlet density exposures that may contribute to subsequent disparities in health outcomes.
